# A sequence-based evolutionary distance method for Phylogenetic analysis of highly divergent proteins

**DOI:** 10.1038/s41598-023-47496-9

**Published:** 2023-11-20

**Authors:** Wei Cao, Lu-Yun Wu, Xia-Yu Xia, Xiang Chen, Zhi-Xin Wang, Xian-Ming Pan

**Affiliations:** Key Laboratory of Ministry of Education for Protein Science, School of Life Sciences, Tsinghua University, Beijing, 100084 China

**Keywords:** Phylogenetics, Protein sequence analyses

## Abstract

Because of the limited effectiveness of prevailing phylogenetic methods when applied to highly divergent protein sequences, the phylogenetic analysis problem remains challenging. Here, we propose a sequence-based evolutionary distance algorithm termed sequence distance (SD), which innovatively incorporates site-to-site correlation within protein sequences into the distance estimation. In protein superfamilies, SD can effectively distinguish evolutionary relationships both within and between protein families, producing phylogenetic trees that closely align with those based on structural information, even with sequence identity less than 20%. SD is highly correlated with the similarity of the protein structure, and can calculate evolutionary distances for thousands of protein pairs within seconds using a single CPU, which is significantly faster than most protein structure prediction methods that demand high computational resources and long run times. The development of SD will significantly advance phylogenetics, providing researchers with a more accurate and reliable tool for exploring evolutionary relationships.

## Introduction

Evolutionary information on protein sequences is crucial for various purposes, including homologue detection^1,2^, protein design, and drug target selection^3^. Phylogenetic analysis is a widely used method for extracting this information that provides valuable insights into the early evolution of proteins, such as identifying ancestral peptide motifs and key sites for protein conformation shifts. However, when dealing with superfamilies containing remote homologues, accurate and unbiased phylogenetic analysis remains a challenge because of high sequence divergence and a large data scale.

A protein superfamily is the largest group of proteins sharing a common ancestor^4^. Proteins in the same superfamily might have highly divergent sequences with sequence identity as low as 15%^5^. Traditional phylogenetic analysis methods, such as Bayesian inference, maximum likelihood (ML), maximum parsimony, and distance-based methods, often perform poorly in the analysis of highly divergent sequences such as those found in a superfamily. This is attributable to the unreliable nature of multiple sequence alignments (MSAs) obtained from these sequences^6^. Among these methods, distance-based methods, such as neighbour joining, are good choices for the phylogenetic analysis of protein sequences in a superfamily because of their ability to circumvent MSA degradation and their scalability for large datasets.

The effectiveness of an evolutionary distance matrix is essential for the success of distance-based phylogenetic analysis. There are three main categories of traditional sequence-based evolutionary distance estimation methods. The first category consists of mathematical model-based algorithms that calculate the percentage of nonidentical amino acids and then correct the probability of multiple substitutions in evolution to estimate the evolutionary distance^7–9^. The second category uses a series of residue substitution matrices to estimate the evolutionary distance. These matrices obtained from large datasets of taxa and alignments are divided into two groups: simple and complex. Simple substitution matrices, such as the Dayhoff^10^, Jones-Taylor-Thomton(JTT)^11^, Whelan and Goldman(WAG)^12^, and Müller-Vingron (MV)^13^ models, ignore site heterogeneity during evolution. However, different sites in protein sequences experience varying evolutionary rates and are characterized by different substitution models because of differences in evolutionary pressures^14^. This led to the development of a complex substitution matrix series^15–18^. Both types of evolutionary distance estimation algorithms rely on MSAs. As the awareness of less informative MSAs for remote homologous proteins has increased, there has been a shift towards the use of pairwise sequence alignments (PSAs) in phylogenetic analysis. The third category of evolutionary distance estimation algorithms is based on PSAs, such as the Needleman–Wunsch (NW) algorithm and all-to-all MMseq2^19,20^.

In this study, we proposed the sequence-based algorithm sequence distance (SD) for evolutionary distance estimation. Unlike existing sequence-based methods, this method leverages the correlation information between sites from position-specific scoring matrices (PSSMs) to construct a feature matrix for further evolutionary distance estimation^21^. This approach, which incorporates the correlation between sites, has successfully predicted protein structures^22^, identified protein–protein interactions^23^, and extracted the evolutionary features of key viral proteins^24^. Our results demonstrate that the SD algorithm can accurately measure the evolutionary distances between remote homologues in protein superfamilies and distinguish evolutionary relationships within and between families, even when the protein sequence identity is less than 20%. Furthermore, the SD algorithm continues to perform effectively when the sequence identity is as low as 10%, whereas other sequence-based methods fail at this level. Under the fact that the protein structure being much more highly conserved throughout evolution than the protein sequence, several structure-based phylogenetic analysis methods are developed. Protein structure prediction tools have achieved remarkable advancements in recent CASP14 and CASP15. However, these methods typically demand a substantial amount of computing resources, including CPUs, GPUs, and even TPUs, which may not be accessible to most researchers. Moreover, for proteins with lengths exceeding 1000 residues, predicting their structures may take several days. In contrast, the SD algorithm can efficiently calculate evolutionary distances for thousands of protein pairs in just a few seconds using a single CPU. Furthermore, the distances derived from the SD algorithm highly correlate with structural similarity, and the topology of evolutionary trees based on the SD algorithm is much more similar to that based on structural evolutionary distances than that based on other methods. In conclusion, the SD algorithm can be widely used for the phylogenetic analysis of remote homologues, especially in cases of high sequence divergence.

## Materials and methods

### The superfamily database

The protein superfamily database was constructed on the basis of the SCOP2 database^5^. SCOP2 is a non-redundant, manually classified database that provides information on the structural and evolutionary relationships between proteins. To evaluate the effectiveness of the evolutionary distance on a superfamily level, which is the largest classification level capable of finding common ancestor sequences, we conducted a series of tests. Initially, we filtered out proteins shorter than 50 amino acids or longer than 500 amino acids. After this screening, 31,725 proteins belonging to 2106 superfamilies remained. Then, we eliminated 715 superfamilies that featured only a single family and 938 superfamilies with fewer than five protein domains to ensure further statistical analysis. Then, 529 superfamilies consisting of 14,108 proteins were used for further database construction (Table 1). For each protein superfamily, we built datasets with sequence identities under different thresholds (0.5, 0.4, 0.3, 0.2, and 0.1) using PISCES^25^.

**Table 1:** The counts of folds, superfamilies, families, and proteins under different sequence identities in the superfamily database.

Sequence identity thresholds	Fold	Superfamily	Family	Protein
0.1	322	401	2714	5741
0.2	324	402	2725	6080
0.3	324	403	2734	7848
0.4	397	529	3065	11,162
0.5	398	529	3093	13,717
All	398	529	3093	14,108

### SD algorithm

The state-of-the-art sequence-based evolutionary distance estimation methods assume that each site in the protein sequence is independent. In the evolutionary distance estimation algorithm SD, we consider the correlations between adjacent sites in protein sequences. The algorithm utilizes the interactions between residues as extracted from PSSMs and considers the predicted secondary structure and solvent accessibility to depict the local structure of sites. Based on these features, a feature profile is constructed. SD then employs PSA using these feature profiles to eliminate low-quality MSAs. The evolutionary distance is ultimately calculated on the basis of the best alignment score.

**Figure 1:**
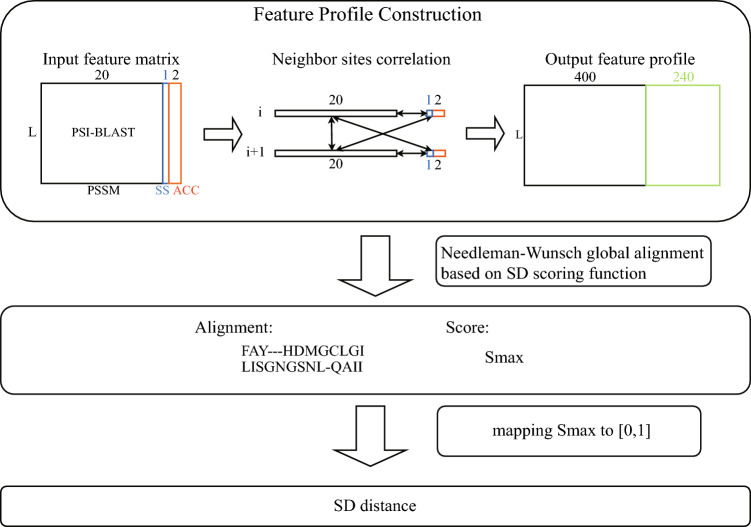
Overview of the SD algorithm.

The SD algorithm uses three input features: the PSSM, predicted secondary structure, and solvent accessibility. The PSSM is calculated using PSI-BLAST in the BLAST v2.2.25 package with a three-iteration search of the Uniref90 database and an E-value threshold of 0.001^26^. Each element in the PSSM represents the probability of occurrence of 20 amino acids at each site in the protein. The secondary structure and solvent accessibility are predicted by SPIDER2^27^. The secondary structure feature is one-dimensional, and it can take on the values H (α-helix), E (β-sheet layer), and C (irregularly coiled). The solvent accessibility feature describes the solvent contact area of the amino acid at the site. This characteristic is assigned values of either B (buried) when the relative accessible surface area (rASA) of the residue predicted by SPIDER2 is less than 20%, or E (exposed) when the rASA exceeds 20%^27^. This resulted in a total of 23 input features for each site, including 20-dimensional amino acid occurrence probability features, 1-dimensional secondary structure features, and 2-dimensional solvent accessibility features (Fig. 1).

The construction of a feature profile is a critical step in the SD algorithm, as it incorporates the correlations between sites into the original input features. This transformation process consists of two main steps: (1) constructing the probability profile of a specific residue pair occurrence at adjacent sites by calculating the cross product of the probability of amino acid occurrence at site i and site i + 1, resulting in a 20 × 20-dimensional vector, and (2) constructing the probability profile of the intersection of neighbouring residue occurrence types and local structural features. The crossover information is obtained as a total of 4 × 3 × 20-dimensional vectors. After these transformation steps, the feature profile for each site is a 640-dimensional vector that considers the correlation between neighbouring sites.

Next, we defined a pairwise scoring function for feature profile alignment, as presented in Eq. (1). Given matching between site i of protein sequence L1 and site j of sequence L2, the scoring function is

1 $$S(i,j)={M}_{L1}(i)\cdot {M}_{L2}(j)+{\omega }_{1}SS(i,j)+{\omega }_{2}rACC(i,j)$$

The SD algorithm calculates the evolutionary distance between two protein sequences by first using a global alignment algorithm based on a scoring function of the site feature profile. The scoring function consists of three terms. ${M}_{L1}$ is the feature profile (L1 × 640-dimentional vector) for protein sequence L1. And the same definition for ${M}_{L2}$. The first term is the dot product of the feature profile vectors of sites i and j when they are matching. The second term is the secondary structure matching score. In this term, if the predicted secondary structure of two sequence sites is the same, then $SS(i,j)=1$; otherwise, $SS(i,j)=0$, and ${\omega }_{1}$ is the weight coefficient. The final term is the relative solvent accessibility. In this term, if the predicted relative solvent accessibility of two sequence sites is the same, then $rACC(i,j)=1$; otherwise, $rACC(i,j)=0$, and ${\omega }_{2}$ is the weight coefficient. ${\omega }_{1}$ and ${\omega }_{2}$ are chosen ranging from 1.0 to 2.0 through experimentation and optimization.

Pairwise sequence alignment in SD is based on the classical NW algorithm^28^, which requires a global sequence alignment from start to end. The gap penalty function that we used is the affine gap penalty function. As presented in Eq. (2), we implement three scoring matrices. The process of state transfer is divided into four steps: match transfer to match, match transfer to open gap, open gap to match (end gap), and open gap to open gap.

$$M(i,j)=max\left\{\begin{array}{c}M(i-1,j-1)+s({x}_{i},{y}_{i}), Match \;{\mathrm{x}}_{\mathrm{i}},{\mathrm{y}}_{\mathrm{i}} \\ {I}_{x}(i-1,j-1)+s({x}_{i},{y}_{i}), Insertion\; in \;x\\ {I}_{y}(i-1,j-1)+s({x}_{i},{y}_{i}), Insertion\; in \;y\end{array}\right.$$

$${I}_{x}\left(i,j\right)=max\left\{\begin{array}{c}M\left(i-1,j\right)+d, Open\; gap\; in\; x\\ {I}_{x}\left(i-1,j\right)+e, Extend\; gap\; in\; x\end{array}\right.$$

2 $${I}_{y}(i,j)=max\left\{\begin{array}{c}M(i,j-1)+d, Open\; gap\; in\; y\\ {I}_{x}(i,j-1)+e, Extend\; gap\; in\; y\end{array}\right.$$

Since the alignment score obtained by the former process represents the closeness of two sequences, it is mapped to the [0,1] space to calculate the evolutionary distance of two protein sequences.

3 $${D}_{sd}=(1/\sqrt{{S}_{max}}-1)/2$$

### Evaluation of evolutionary distances

To assess the ability of evolutionary distances to identify SCOP relationships at the superfamily level, we introduce the concept of the recognition rate (RR). The RR refers to the percentage of superfamilies for which proteins belonging to the same family and those from different families can be differentiated based on a specified evolutionary distance. First, we calculated the superfamily standard deviation (*sfsd*) as follows:

4 $$sfsd=std\left({d}_{i,j}\right)\;i\in {f}_{m},j\in {f}_{n},m\ne n$$

where $i,j$ are proteins in the same superfamily but in different families and ${d}_{i,j}$ is the evolutionary distance between $i$ and $j$ calculated by different algorithms. $sfsd$ can detect the heterogeneity of distant evolutionary relationships, preventing the omission of situations in which the evolutionary distance between families exceeds the threshold. We used the Mann–Whitney U test to determine whether the distribution of evolutionary distance within the same family and that between different families were significantly different. The RR is defined as follows:

5 $$Q=sf\left(p-value<0.001\right)$$

6 $$RR=\frac{\#Q}{\#sf}$$

where $Q$ is a set of protein superfamilies in which the distribution of evolutionary distance between proteins within the same family is significantly different from the distribution of evolutionary distance between proteins in different families, $\#Q$ is the number of elements in the set, and $\#sf$ represents the total number of superfamilies in the database. The performance of the evolutionary distance in recognizing SCOP relationships increases as the RR increases.

Furthermore, we define relative distance (RD) as follows:

7 $$RD=\frac{1}{\#Q}\sum_{i}\frac{{d}_{diff}^{i}-{d}_{same}^{i}}{{d}_{diff}^{i}}\left(i\in Q\right)$$

RD measures the extent to which evolutionary distances vary within and between families. ${d}_{same}^{i}$ refers to the mean evolutionary distance of proteins within the same family in superfamily *i*, and ${d}_{diff}^{i}$ refers to the mean evolutionary distance between proteins of different families in superfamily *i*. A higher RD indicates better performance of the evolutionary distance.

### Protein similarity calculation

Remote homologues are characterized by low sequence identity but certain structural similarities. Thus, the evolutionary distance used for the phylogenetic analysis of proteins with remote homologues might exhibit a relationship with structural similarities. We used our superfamily database to investigate the correlation between evolutionary distances based on sequence information and structural similarity.

Because proteins with sequence identities greater than 20% are likely to have similar structures, we removed redundant proteins with sequence identities exceeding 20% using the PISCES program^25^. In total, 7035 proteins were retained.

To measure structural similarity, we used the template modelling score (TM-score), which provides a global measure of the structural similarity between proteins^29^.

8 $$TM-score=\frac{1}{N}{\left[\sum_{i=1}^{{N}_{ali}}\frac{1}{1+\left({D}_{i}^{2}/{D}_{0}^{2}\right)} \right]}_{max}$$

9 $${D}_{0}=1.24\sqrt[3]{N-15}-1.8$$

In the formulas, ${D}_{0}$ denotes the scale factor, which makes the TM-score length-independent. ${D}_{i}$ is the distance of the i-th pair of the equivalent residues between the two structures, which depends on the superposition matrix; the ‘max’ means the procedure to identify the optimal superposition matrix that maximizes the sum in Eq. 8^29^. $N$ is the sequence length of the template protein. ${N}_{ali}$ is the length of the aligned sequence. To obtain a more appropriate expression of the structural similarity between two protein sequences, it is important to normalize the TM-score based on the appropriate chain. As different chains can result in different values, we use the following formula to calculate the TM-score:

10 $${TM-score}_{i,j}=\frac{{N}_{i}{TM}_{i}+{N}_{j}{TM}_{j}}{{N}_{i}+{N}_{j}}$$

Here, ${N}_{i}$ refers to the length of protein sequence $i$, and ${TM}_{i}$ represents the calculated TM-score normalized by protein sequence $i$. The same definition applies to ${N}_{j}$ and ${TM}_{j}$. Formula (10) ensures a more accurate reflection of structural similarity between two protein sequences, irrespective of the chain used for normalization.

### Phylogenetic tree construction

In this study, several sequence-based evolutionary distance algorithms were implemented for comparison. They are mainly from three classes. The first class consists of mathematical model-based evolutionary distances. The Raw distance, JCP distance, Kimura distance, and Scoredist distance were calculated by Belvu 2.26, which require the input of MSAs obtained from Cluster Omega^30^. The second class includes residue substitution matrix-based evolutionary distances, which are divided into simple substitution matrix-based distances and complex substitution matrix-based distances. The former include the EXP-DAY, EXP-JTT, EXP-MV, EXP-WAG, ML-DAY, ML-JTT, ML-MV, and ML-WAG distances calculated by lapd 1.0^31^. The latter type is the IQ-Tree-ML distance, which is calculated by IQ-Tree v1.6.9^32^ using different complex amino acid substitution matrices and selecting the optimal matrix by the ML rule. The MSA was obtained from Cluster Omega^30^. The third class includes pairwise sequence alignment-based evolutionary distance. The Needleman–Wunsch (NW) distance was calculated using the biopython package^33^, whereas the MMseq2 distance was obtained directly from the intermediate results generated by the evolutionary analysis software graph splitting^19^. For phylogenetic tree construction, we used BioNJ, an accurate and widely used evolutionary distance-based phylogenetic tree generation method^34^.

### Evaluation of the accuracy of the phylogenetic tree

We calculated the Robinson–Foulds (RF) distance to evaluate the accuracy of the estimated phylogenetic tree using ETE3 3.1.2^35^. The RF distance between two trees is defined as the number of steps required to convert the first tree topology into the second tree topology. This process involves interrupting the branch (edge) unique to tree A and generating a branch unique to tree B. Consequently, the RF distance between trees A and B is equal to (n1 + n2), where n1 represents the number of branches unique to tree A and n2 represents the number of branches unique to tree B. There are algorithms that normalize the RF distance. The advantages of the RF distance are simplicity, intuitiveness, and low computational cost.

## Results

### The evolutionary distance calculated by SD is highly correlated with protein structural similarity

For proteins in constructed superfamily database with varying sequence identities, we investigated the relationship between the structural similarity score (TM-score) and the evolutionary distance. Specifically, we compared the SD distance with the NW distance, MMseq2 distance and IQ-Tree distance. As detailed in the Materials and Methods, NW, SD and MMseq2 are based on PSA, while the IQ-Tree distance is based on MSA.

**Figure 2:**
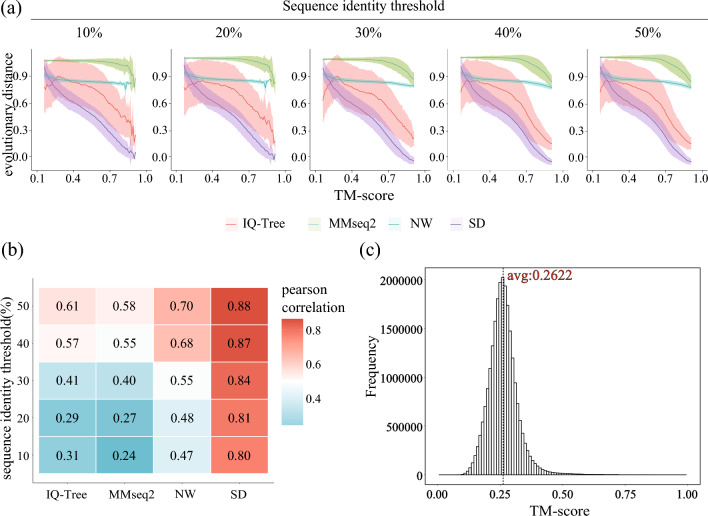
Correlation between the evolutionary distance and structural similarity. (**a**) The correlation between evolutionary distances and TM-score with varying sequence identities. The line reflects the mean distance, and the shadow reflects the standard deviation. (**b**) The absolute values of the Pearson correlation were calculated for each distance under a series of sequence identity thresholds. (**c**) The distribution of TM-scores in the superfamily dataset.

Protein pairs within each superfamily were pooled and analysed under different thresholds to calculate evolutionary distances and TM-scores. They were divided into groups based on their TM-scores, and the mean and variance of different evolutionary distances were calculated for each group (Fig. 2a).

As illustrated in Fig. 2b,c, in the superfamily dataset, the mean of TM-score is 0.2622, and the Pearson correlation between evolutionary distances and TM-score increased with increasing sequence identity. However, for proteins with low sequence identity (10% and 20%), the SD distance could effectively reflect the evolutionary distances of protein structures while other evolutionary distances failed. The PSA-based distances performed better than MSA-based distances. IQ-Tree distance displayed a large variance in each TM-score-based group, while MMseq2 distance barely had variance in the 0.1 to 0.5 TM-score range. SD decreased smoothly with increasing TM-scores; conversely, the NW distance displayed a noticeable decrease only in the initial section, and its mean value remained unchanged when the TM-score exceeded 0.4. For protein pairs with a sequence identity under 20%, the overall Pearson correlation between the SD evolutionary distance and TM-score was −0.80867, and the correlation between the NW evolutionary distance and TM-score was −0.47572. A larger SD evolutionary distance indicates lower structural similarity between proteins, whereas other PSA-based evolutionary distances are limited in its ability to reflect changes in protein structural similarity. Meanwhile, the SD distance provided a better indication of the variation in protein structural similarity and has the potential to be applied to the search for distant templates.

In this study, we compared SD with the widely used protein distant homologue identification software SPARKS-X and HHblits, which incorporate template structural information^36,37^. The SCOP20_v1.75 database contains 6365 proteins with less than 20% sequence identity. We evaluated the similarity of the structures of the Top1 distant homologues identified by different methods to the target proteins, measured by the TM-score.

For SPARKS-X, the profile of each sequence was obtained by three iterations of PSI-BLAST (with an E value threshold of 0.001), and for HHblits, directly from https://github.com/soedinglab/hh-suite. All parameters were set as default for SPARKS-X and HHblits. As shown in Supplementary Table 1, the mean TM-scores of the Top1 distant homologues identified by SPARKS-X, HHblits, and SD were 0.6216, 0.6199, and 0.61538, respectively. Additionally, the percentages of proteins with TM-scores > 0.5 were 75.75%, 76.99%, and 76.10%, respectively. The performance of SD was comparable to that of SPARKS-X and HHblits for both evaluation criteria.

### The SD algorithm can accurately distinguish SCOP relationships at the superfamily level

To evaluate the performance of the SD algorithm in distinguishing evolutionary relationships within and between families at the superfamily level, we calculated the RR and RD and compared these parameters with those calculated using other evolutionary distance methods. The details of the RR and RD calculations can be found in the Materials and Methods.

**Figure 3:**
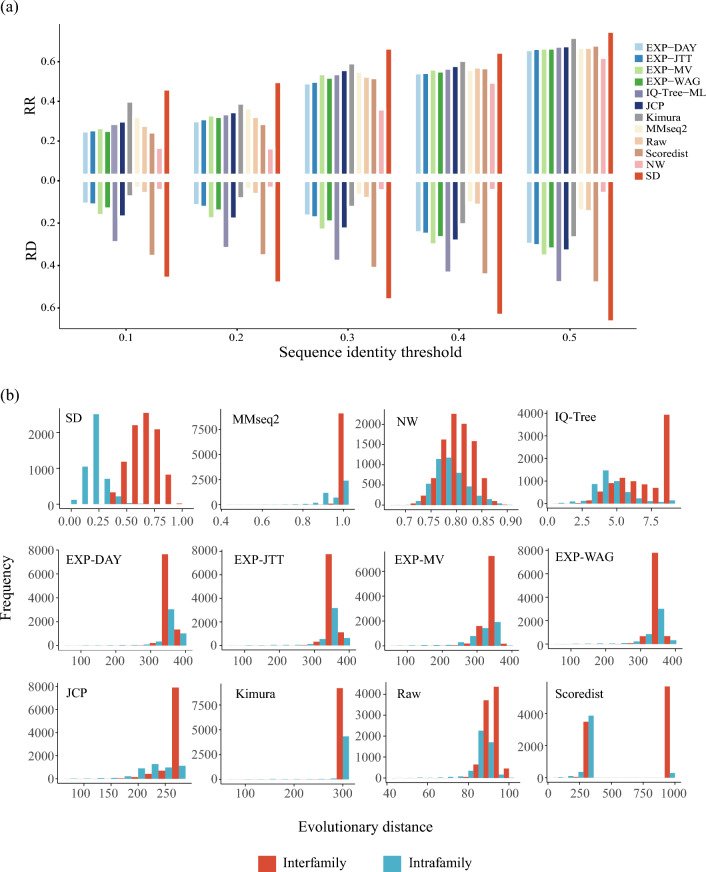
The SD algorithm can effectively distinguish evolutionary relationships at superfamily level. (**a**)Performance of evolutionary distance methods under different homology thresholds. SD outperformed other sequence-based evolutionary distance methods at each threshold, and this superiority remained even under low sequence identity. (**b**) Distribution of distinguished evolutionary relationships by SD distance and other evolutionary distances in the Homeodomain-like superfamily based on PSA (MMseq2 distance, NW distance), complex amino acid substitution matrix (IQ-Tree distance), simple amino acid substitution matrix (EXP-Dayhoff, EXP-WAG, EXP-JTT, EXP-MV), mathematical correction model (Raw distance, JCP distance, Kimura distance, Scoredist distance) methods.

As illustrated in Fig. 3a, evolutionary relationships were more easily detected when sequence homology exceeded 0.3. To evaluate the ability of evolutionary distances to distinguish proteins within and between families, different sequence homology thresholds were set. Our results demonstrated that the SD algorithm performed best in terms of the RR and RD when the homology threshold ranged from 0.1 to 0.5. As the homology threshold decreased, both the RR and RD decreased for all evolutionary distances tested. Under a homology threshold of 0.5, the SD algorithm demonstrated significant differences in evolutionary distances between and within families for 75.24% of superfamilies, with an RD of 0.66. Under homology thresholds of 0.4 and 0.3, the SD algorithm still performed best, with significant differences in evolutionary distances between and within families measured for 64.69% and 66.75% of superfamilies, respectively. The slight superiority of SD under the 0.3 sequence identity threshold compared to the 0.4 threshold can primarily be attributed to variations in dataset distribution across these different sequence identity thresholds. Furthermore, it is important to consider that RR inherently demonstrates statistical variability, which can lead to fluctuations in performance metrics when assessed across varying thresholds. However, these results were generally inferior to those obtained under a homology threshold of 0.5.

In the twilight zone of protein sequence evolution, distinguishing evolutionary relationships within and between families at the superfamily level is challenging. At a homology threshold of 0.2, compared to a threshold of 0.3, although proteins in the same family generally maintain similar conformations or functions, their recognition ability rapidly weakens, causing a sharp drop in the RR from 66.75% to 49.86%. RD also decreased by approximately 14% from 0.5606 at the 0.3 homology threshold to 0.4811 at a threshold of 0.2. This decrease in performance is mainly attributed to an increase in the evolutionary distance within the family as the sequence homology decreases. Despite these challenging conditions, the SD algorithm still outperformed other evolutionary distances. The performance of the SD algorithm at the superfamily level was further demonstrated by its stability even at a low homology threshold of 0.1. The RR only decreased from 49.86% to 46.11%. Similarly, this gap was also reflected in RD. The downwards trend of the two indicators tended to be flat. Detailed results can be found in Supplementary Table 2.

Compared to other evolutionary distance methods, SD demonstrated superior performance for both the RR and RD, which was maintained at different homology thresholds. The SD algorithm's advantages are particularly evident when sequence homology is high, highlighting its broad discrimination capabilities. Under the condition of low sequence homology, SD remains applicable. Overall, these results demonstrated that SD can effectively distinguish evolutionary relationships within and between families in the superfamily database, even under conditions of low sequence identity.

### Case study: SD distance of the Homeodomain-like superfamily

We analysed the performance of SD in determining evolutionary relationships from the perspective of the entire superfamily database. In this section, we utilized the Homeodomain-like superfamily, which consists of proteins with helices involved in DNA binding^38,39^, as a case study to further evaluate the ability of SD to infer evolutionary relationships. With a sequence identity threshold of 50%, the Homeodomain-like superfamily comprises 167 proteins divided into 24 families. Notably, the Tetracyclin repressor-like family has more than 50 domains, while two other families have more than 20 domains, and six families have more than two domains (see Supplementary Table 3). These characteristics of the Homeodomain-like superfamily highlight its sequence variability and evolutionary diversity. To illustrate the actual distribution of evolutionary distances within the Homeodomain-like superfamily, we calculated pairwise alignment-based, mathematical model-based and residue substitution matrix-based evolutionary distances of proteins in this superfamily (Fig. 3b).

Several methods for measuring evolutionary relationships, such as PSA or complex substitution matrix-based methods, exhibit varying degrees of accuracy. The NW method provides an effective measure of relationships that occur within and between families, but it displays significant overlap between the distance distributions of interfamily and intrafamily relationships, indicating its inability to differentiate between close and distant relationships (also known as the twilight zone). Conversely, IQ-Tree performs well in depicting intrafamily evolutionary relationships, whereas most interfamily distances take a maximum value. Measurement of the latter was beyond the scope of IQ-Tree. Similarly, MMseq2 also fails to accurately measure the evolutionary relationships between families. In contrast, SD distinguishes between close and distant evolutionary relationships effectively, with distances measured by SD tending to be larger for relationships between families than for relationships within families. Furthermore, the distribution of distances of the two groups were significantly different (p < 0.001, Mann–Whitney U test).

Although the R distance calculation does not involve sequence alignments, it can measure remote evolutionary relationships. However, there is overlap between interfamily and intrafamily distance distributions. As shown in Fig. 3b, JCP distance, Kimura distance, and Scoredist distance fail to effectively distinguish remote evolutionary relationships, with most interfamily relationships assigned a maximum value of 300. The findings indicate that the effective range of the existing mathematical correction-based evolutionary distances is insufficient for accurately estimating the evolutionary distances of distant sequences within protein superfamilies. Additionally, the quality of MSA may be compromised when incorporating highly divergent sequences, resulting in a failure to measure close evolutionary relationships.

Simple amino acid substitution-based models perform well in inferring proximate relatives, but their range is limited in measuring remote evolutionary relationships. EXP-DAY, EXP-JTT, and EXP-WAG underperform in this regard, whereas EXP-MV is capable of estimating remote evolutionary relationships. However, upon a comparison of evolutionary distances within and between families, the distributions of the two groups are similar, indicating that these evolutionary distances are inadequate in distinguishing between close and distant evolutionary relationships.

### Performance of SD distances for the phylogenetic analysis of superfamilies

In this section, we utilized the SD distance to perform phylogenetic analysis of protein sequences within a superfamily. Specifically, phylogenetic trees were constructed using SD distances, and their topological features were compared to those of trees constructed using the TM-score as a structural evolutionary distance. The TM-score, which represents structural similarity, is more suitable for evolutionary tree construction than sequence homology since two distant homologous proteins with insignificant sequence similarity could adopt a common fold and may perform similar biochemical functions^40^. The accuracy of the phylogenetic trees was assessed by comparing their topologies with that of the evolutionary tree generated by BioNJ based on the TM-score, and the RF distance was used to measure topology similarity, where smaller distances indicate greater accuracy.

**Figure 4:**
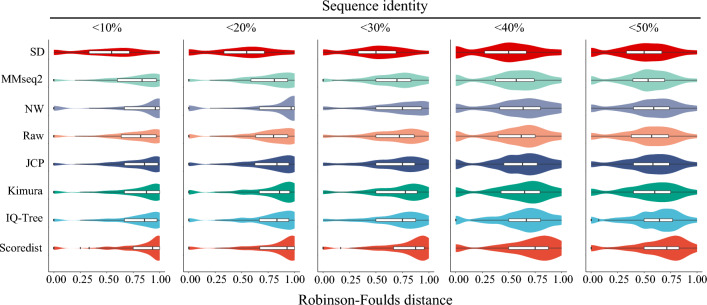
The distribution of RF distances between the evolutionary distance-based phylogenetic trees and the TM-score-based phylogenetic trees analysed under varying sequence identities.

Our results demonstrated that the RF distances of the SD phylogenetic tree ranged mainly from 0.25 to 0.7, with a probability of 0.1189 that the RF distance would be 0, indicating that in 11.89% of superfamilies, the SD-based phylogenetic trees had the exact same topology as the TM-score-based phylogenetic tree (Fig. 4). Moreover, under different sequence identity thresholds, SD consistently displayed a lower RF distance than the other methods. Notably, the mean RF distance hardly changes with increasing sequence identity, suggesting that SD is less influenced by sequence identity. Taken together, our findings highlight the suitability of SD for the phylogenetic analysis of proteins within superfamilies.

### Case study: Phylogenetic analysis of the Flavoreductase-like superfamily

**Figure 5:**
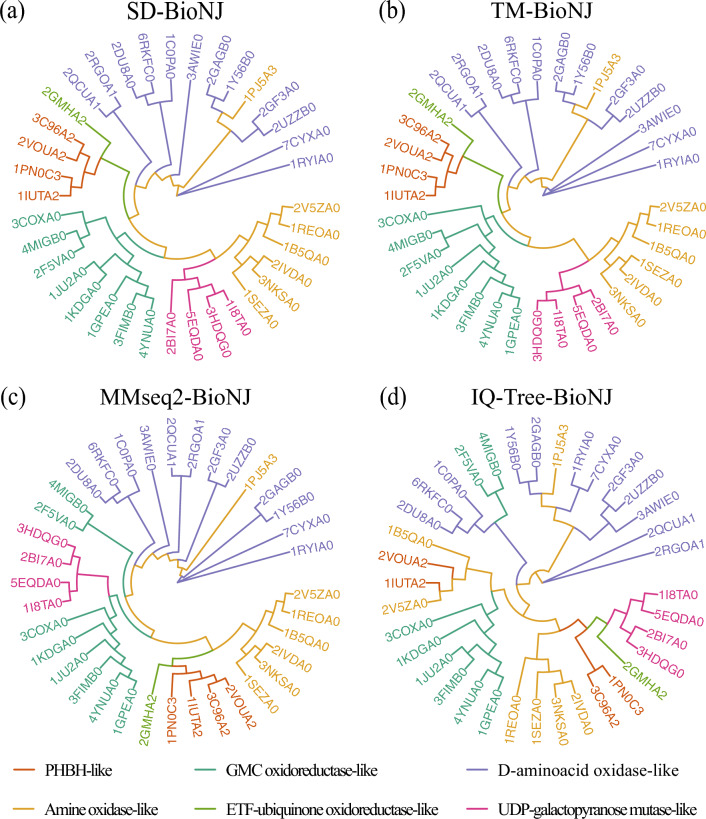
Topology of different evolutionary trees for the 3,000,055 superfamily. The colour-coded branches denote distinct families. (**a**) The phylogenetic tree generated using SD. (**b**) The reference evolutionary tree constructed on the basis of the structural evolutionary distance TM-score. (**c**) The evolutionary tree derived from pairwise sequence alignment-based evolutionary distance MMseq2. (**d**) The phylogenetic tree obtained using the ML method in IQ-Tree.

The Flavoreductase-like superfamily (ID: 3,000,055) is an important group of proteins that function as FAD/NAD(P) binders^41^, and it comprises 36 proteins with sequence identities under 50% (see Supplementary Table 4). To gain a deeper understanding of the evolutionary relationships between the six distinct families within this superfamily, we conducted a comprehensive analysis. The SD algorithm required 21 s on a single CPU to calculate the distance matrix for the set of 630 protein pairs. We constructed phylogenetic trees based on various evolutionary distances and compared them to the TM-score-based tree. Our results indicated that the SD-BioNJ phylogenetic tree was the most similar to the TM-score-based tree among all the trees based on different algorithms (Fig. 5). This observation suggests that the SD distance is a reliable metric for measuring evolutionary distances among the members of this superfamily. Both the SD-BioNJ and TM-BioNJ trees grouped the GMC oxidoreductase-like, UDP-galactopyranose mutase-like and Amine oxidase-like protein families together, indicating their close evolutionary relationships, and only a few minor variations were observed in the evolutionary relationships within families. In terms of overall topological differences, the SD-BioNJ tree had the lowest RF distance of 0.212 when compared to the reference tree, while the IQ-Tree-BioNJ tree and MMseq2-BioNJ tree had higher RF distances of 0.606 and 0.424, respectively.

Therefore, our findings suggest that the SD distance can effectively distinguish the evolutionary relationships between families within the Flavoreductase-like superfamily and that the SD-BioNJ tree is a useful tool for the phylogenetic analysis of protein sequences in this superfamily. Our detailed analysis of the Flavoreductase-like superfamily provides valuable insights into the evolutionary relationships between its six different families. Topology of different evolutionary trees for the Homeodomain-like superfamily can be found in Supplementary Fig. S1. The SD distance metric was found to be the most accurate among the evolutionary distance-based methods for phylogenetic analysis of this superfamily.

## Discussion

We developed the SD algorithm for protein evolutionary distance calculation based on a single protein sequence. Since the evolutionary processes of different sites are influenced by the local structure and amino acids of the surrounding sites, we extracted the interaction information from neighbouring residues using a PSSM and utilized the predicted secondary structure information and solvent accessibility information to depict local structure. In addition, pairwise sequence alignment was performed using the SD algorithm to avoid the impact of low-quality MSAs.

In remote evolutionary relationship analysis, SD outperformed traditional methods, accurately measuring inter- and intrafamily evolutionary distances in the SCOP2 database, especially under low sequence homology thresholds. We found that SD was more effective than other methods in distinguishing intra- and interfamily proteins under different homology thresholds, as demonstrated by the RR and RD. As the SD distance correlates well with protein structural similarity and can identify a comparable percentage of distant homologues in the SCOP20 dataset, it can be applied to search for distant homologous sequences. When combined with structural information, it supports an effective distant homologous template search in protein structure prediction.

In terms of phylogenetic analysis, the SD algorithm yielded high topological similarity with evolutionary trees constructed on the basis of structural similarity. We measured the difference in topology between trees using the RF distance metric and found that the mean RF distance between SD-BioNJ trees and structural similarity-based trees was smaller than those of IQ-Tree-BioNJ and MMseq2-BioNJ trees. A case study of the Flavoreductase-like superfamily demonstrated that the SD-based tree and TM-score-based tree depicted similar evolutionary relationships among protein families, but they differed in describing some proteins within families. In terms of computational efficiency, we evaluated the SD algorithm on several randomly selected sets of protein sequences from the superfamily database, and it requires only a few seconds on a single CPU to compute the distance matrix for thousands of protein pairs (Supplementary Table 5).

The increasing number of protein structures solved through X-ray crystallography and nuclear magnetic resonance (NMR) techniques has highlighted the fact that the protein structure being much more highly conserved throughout evolution than the protein sequence. This is because tertiary structures are subject to strict constraints during evolution to maintain structural stability, functional integrity, and folding correctness under the pressure of natural selection. In fact, the evolution rate of protein structures is significantly lower than that of protein sequences. New folds emerge on a timescale of billions of years^42^. Several approaches have been taken to incorporate structural information into evolutionary analysis. A stochastic evolution model that combines an insertion/deletion model, an amino acid substitution model, and a structural drift model has been proposed^43^. Another group used an amino acid substitution model that considers both amino acid identity and side-chain conformational states to estimate evolutionary distances^44^. Protein structural alignment algorithms, including Q-score and RMSD, are also used^45,46^. Incorporating structural information can potentially enhance the accuracy of evolutionary distance estimation. However, despite the significant progress made in computational protein structure prediction methods in recent years, their accuracy is still limited and they often require high computational resources and long execution times. Consequently, the practical application of structure-based algorithms for evolutionary distance estimation may be constrained.

### Supplementary Information

Supplementary Information.

## Data Availability

The SD program has been written in C++ language, and it runs on the Linux platform. It is free for use on the web server http://166.111.152.74:8888/sd_distance/. For academic users, the executable code and superfamily database can be obtained by e-Mail: pan-xm@mail.tsinghua.edu.cn.
