# Publisher Correction: A sequence-based evolutionary distance method for Phylogenetic analysis of highly divergent proteins

**DOI:** 10.1038/s41598-023-50520-7

**Published:** 2023-12-28

**Authors:** Wei Cao, Lu-Yun Wu, Xia-Yu Xia, Xiang Chen, Zhi-Xin Wang, Xian-Ming Pan

**Affiliations:** Key Laboratory of Ministry of Education for Protein Science, School of Life Sciences, Tsinghua University, Beijing, 100084 China

Correction to: *Scientific Reports* 10.1038/s41598-023-47496-9, published online 20 November 2023

The original version of this Article contained an error in Figure 3, panel b, where the labels are not displayed.

The incorrect Figure 3 and the accompanying legend appear below.

**Figure 3:**
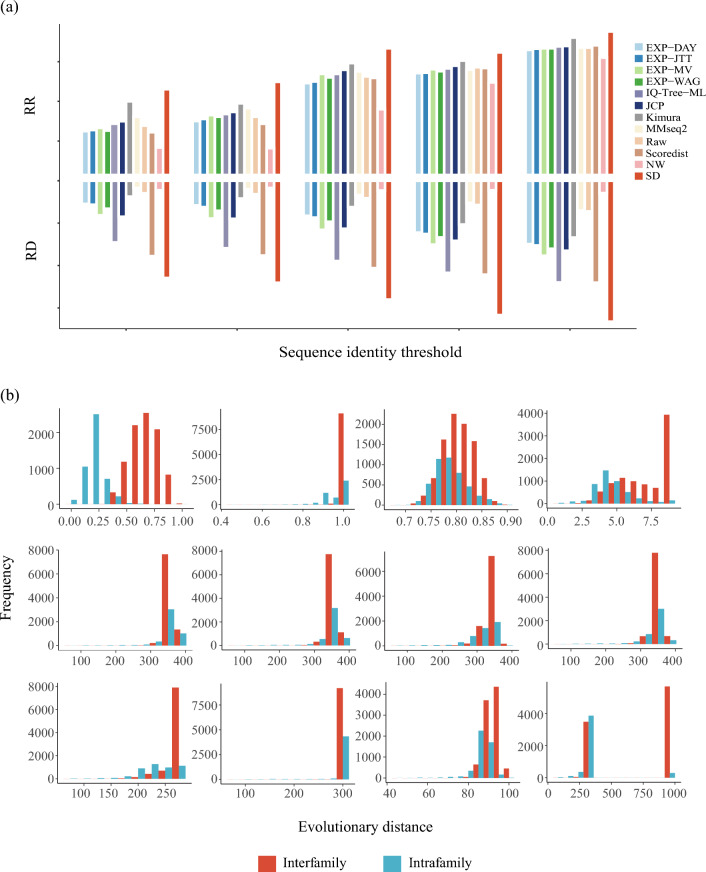
The SD algorithm can effectively distinguish evolutionary relationships at superfamily level. (**a**) Performance of evolutionary distance methods under different homology thresholds. SD outperformed other sequence-based evolutionary distance methods at each threshold, and this superiority remained even under low sequence identity. (**b**) Distribution of distinguished evolutionary relationships by SD distance and other evolutionary distances in the Homeodomain-like superfamily based on PSA (MMseq2 distance, NW distance), complex amino acid substitution matrix (IQ-Tree distance), simple amino acid substitution matrix (EXP-Dayhoff, EXP-WAG, EXP-JTT, EXP-MV), mathematical correction model (Raw distance, JCP distance, Kimura distance, Scoredist distance) methods.

The original Article has been corrected.

