# Supplementary material for: A sequence-based evolutionary distance method for Phylogenetic analysis of highly divergent proteins
Source: Sci Rep. 2023 Nov 20;13:20304. doi: 10.1038/s41598-023-47496-9 (PMC10662474; doi:10.1038/s41598-023-47496-9)
Supplement: Supplementary file 1 — Supplementary Information. [file 41598_2023_47496_MOESM1_ESM.docx]

**A sequence-based evolutionary distance method for Phylogenetic analysis of highly divergent proteins**

Wei Cao^1^**,** Lu-Yun Wu^1^, Xia-Yu Xia^1^, Xiang Chen, Zhi-Xin Wang* and Xian-Ming Pan*

Key Laboratory of Ministry of Education for Protein Science, School of Life Sciences, Tsinghua University, Beijing 100084, China.

*To whom correspondence should be addressed: Dr. Xian-Ming Pan, or Dr. Zhi-Xin Wang

School of Life Sciences, Tsinghua University, Beijing, 100084, China, Telephone: +86-10-62792827; E-Mail: pan-xm@mail.tsinghua.edu.cn

^1^ authors with equal contributions

**Supplementary Materials**

**Supplementary Table 1** Top1 TM-score of homologous protein detection in the SCOP20_v1.75 database.

| SPARKS-X | | HHblits | | SD | |
| --- | --- | --- | --- | --- | --- |
| Top1 TM | TM>0.5 | Top1 TM | TM>0.5 | Top1 TM | TM>0.5 |
| 0.6216 | 75.75% | 0.6199 | 76.99% | 0.61538 | 76.10% |

**Supplementary Table 2** Performance of evolutionary distance methods under different homology thresholds

| Distance | 0.1 | | 0.2 | | Threshold  0.3 | | 0.4 | | 0.5 | |
| --- | --- | --- | --- | --- | --- | --- | --- | --- | --- | --- |
|  | RR | RD | RR | RD | RR | RD | RR | RD | RR | RD |
| SD | **46.11%** | **0.4584** | **49.86%** | **0.4811** | **66.75%** | **0.5606** | **64.69%** | **0.6338** | **75.24%** | **0.6648** |
| Raw | 27.78% | 0.0590 | 32.25% | 0.0633 | 52.58% | 0.0825 | 57.20% | 0.1144 | 67.12% | 0.1447 |
| JCP | 30.00% | 0.1697 | 34.69% | 0.1796 | 55.93% | 0.2262 | 57.99% | 0.2835 | 67.89% | 0.3301 |
| Kimura | 40.00% | 0.0746 | 39.02% | 0.0836 | 59.28% | 0.1242 | 60.55% | 0.2064 | 72.15% | 0.2679 |
| Scoredist | 24.44% | 0.3555 | 28.73% | 0.3528 | 51.80% | 0.4123 | 56.80% | 0.4430 | 68.28% | 0.4807 |
| EXP-DAY | 25.00% | 0.1092 | 30.08% | 0.1163 | 49.23% | 0.1653 | 54.24% | 0.2446 | 65.96% | 0.2989 |
| EXP-JTT | 25.56% | 0.1129 | 31.17% | 0.1245 | 50.00% | 0.1742 | 54.44% | 0.2512 | 66.54% | 0.3049 |
| EXP-MV | 26.67% | 0.1629 | 33.06% | 0.1781 | 53.87% | 0.2315 | 56.21% | 0.3016 | 66.73% | 0.3538 |
| EXP-WAG | 25.28% | 0.1318 | 32.25% | 0.1416 | 52.06% | 0.1932 | 56.61% | 0.2676 | 66.73% | 0.3205 |
| ML-DAY | 31.11% | 0.2063 | 35.23% | 0.2164 | 56.70% | 0.2738 | 57.00% | 0.3456 | 67.31% | 0.3996 |
| ML-JTT | 28.89% | 0.2093 | 34.49% | 0.2245 | 56.44% | 0.2810 | 56.80% | 0.3487 | 67.12% | 0.3973 |
| ML-MV | 29.17% | 0.2504 | 34.15% | 0.2701 | 55.41% | 0.3186 | 56.02% | 0.3771 | 67.31% | 0.4208 |
| ML-WAG | 30.00% | 0.2241 | 36.59% | 0.2392 | 55.67% | 0.2941 | 56.80% | 0.3597 | 67.12% | 0.4077 |
| IQ-Tree | 28.06% | 0.2909 | 33.60% | 0.3185 | 53.87% | 0.3788 | 56.61% | 0.4345 | 67.70% | 0.4796 |
| MMseq2 | 32.22% | 0.0342 | 36.59% | 0.0395 | 55.15% | 0.0677 | 56.02% | 0.1047 | 67.12% | 0.1404 |
| NW | 16.70% | 0.0443 | 16.40% | 0.0340 | 36.03% | 0.0461 | 49.55% | 0.0446 | 62.06% | 0.0581 |

**Supplementary Table 3** Distribution of protein sequence numbers in the Homeodomain-like superfamily

| Number of domains | Number of families |
| --- | --- |
| >50 | 1 |
| 21–50 | 2 |
| 6–20 | 0 |
| 2–5 | 6 |
| 1 | 15 |

**Supplementary Table 4** Proteins in Flavoreductase-like superfamily

| Family ID (SCOP) | Function | Protein number |
| --- | --- | --- |
| 4000122 | GMC oxidoreductase-like | 8 |
| 4000123 | PHBH-like | 4 |
| 4000124 | d-amino acid oxidase-like | 12 |
| 4000125 | UDP-galactopyranose mutase-like | 4 |
| 4000127 | ETF-ubiquinone oxidoreductase-like | 1 |
| 4000128 | Amine oxidase-like | 7 |

**Supplementary Table 5** Average calculation time for evolutionary distances of a set of randomly selected proteins using a single CPU over 100 trials.

| Protein number | Protein pairs | Time |
| --- | --- | --- |
| 10 | 45 | 0.72 |
| 20 | 190 | 2.56 |
| 50 | 1225 | 15.02 |
| 100 | 4950 | 59.85 |
| 200 | 19900 | 235.71 |

**Supplementary Figure 1** Topology of different evolutionary trees for the Homeodomain-like superfamily. The colour-coded branches denote distinct families. a) The phylogenetic tree generated using SD. b) The reference evolutionary tree constructed on the basis of the structural evolutionary distance TM-score. c) The evolutionary tree derived from pairwise sequence alignment-based evolutionary distance MMseq2. d) The phylogenetic tree obtained using the ML method in IQ-Tree.
